# Remaining Useful Life Estimation of MoSi_2_ Heating Element in a Pusher Kiln Process

**DOI:** 10.3390/s24051486

**Published:** 2024-02-24

**Authors:** Hafiz M. Irfan, Po-Hsuan Liao, Muhammad Ikhsan Taipabu, Wei Wu

**Affiliations:** Department of Chemical Engineering, National Cheng Kung University, Tainan 70101, Taiwan; irfan.riasat@yahoo.com (H.M.I.);

**Keywords:** generative adversarial network, predictive maintenance, machine learning, Remaining Useful Life, Long Short-Term Memory, Support Vector Regression

## Abstract

The critical challenge of estimating the Remaining Useful Life (RUL) of MoSi2 heating elements utilized in pusher kiln processes is to enhance operational efficiency and minimize downtime in industrial applications. MoSi2 heating elements are integral components in high-temperature environments, playing a pivotal role in achieving optimal thermal performance. However, prolonged exposure to extreme conditions leads to degradation, necessitating precise RUL predictions for proactive maintenance strategies. Since insufficient failure experience deals with Predictive Maintenance (PdM) in real-life scenarios, a Generative Adversarial Network (GAN) generates specific training data as failure experiences. The Remaining Useful Life (RUL) is the duration of the equipment’s operation before repair or replacement, often measured in days, miles, or cycles. Machine learning models are trained using historical data encompassing various operational scenarios and degradation patterns. The RUL prediction model is determined through training, hyperparameter tuning, and comparisons based on the machine-learning model, such as Long Short-Term Memory (LSTM) or Support Vector Regression (SVR). As a result, SVR reflects the actual resistance variation, achieving the R-Square (R^2^) of 0.634, better than LSTM. From a safety perspective, SVR offers high prediction accuracy and sufficient time to schedule maintenance plans.

## 1. Introduction

“Industry 4.0” was initially introduced in 2011 during the Hannover Messe, recognized as the most significant industrial technology expo globally. This event, held in Germany, marked the inception of the fourth industrial revolution, which is currently in its early stages of development [1]. Industry 4.0 incorporates two primary dimensions of advancement: the Cyber–Physical System (CPS), a comprehensive control system that integrates networks, computational capabilities, sensors, and physical objects, and the smart factory, which leverages the CPS system and the Internet of Things (IoT) to enhance manufacturing production, intelligent machinery [2], logistics supply chains [3], human–computer interaction [4], and automated control within factories [5]. The differences between the existing industries and the 4.0 model can be categorized into 3 primary domains: components that possess self-awareness and self-predictive capabilities; machines that exhibit self-awareness, self-predictive abilities, and the ability to self-compare; and a productive system that can self-configure, self-maintain, and self-organize [6].

A push-plate sintering furnace is a type of industrial furnace used for the sintering process, which is a heat treatment method used to compact and harden a material without melting it. This process is commonly used in the production of ceramics, powders, metal powders, and other materials [7]. The furnace is equipped with heating elements that generate heat. These elements are responsible for raising the temperature inside the furnace to the desired sintering temperature. Every single heating element material has its temperature limitation. Heating element problems in a push-plate sintering furnace can significantly impact its performance and efficiency [8]. Therefore, predicting the Remaining Useful Life (RUL) of heating elements (MoSi2) is important to avoid possible machinery failure in a pusher kiln process.

The fundamental attributes of Industry include the capacity for data customization and accessibility, enabling human and automated actions [9]. Data acquisition and analysis play a crucial role in the current period, enabling the development of information that can be utilized for anticipatory purposes or to facilitate collaborative efforts in making predicted decisions. This work was prompted by the concept that the manufacturing industry should transition toward predictive manufacturing [10]. Predictive Maintenance (PdM) is a methodology that relies on historical data, models, and domain expertise. Using statistical or machine-learning models enables anticipating forthcoming problems, enhancing the decision-making process for maintenance activities. This predictive capability identifies trends, behavior patterns, and correlations, minimizing downtime and improving overall operational efficiency [11]. Machine learning, an interdisciplinary field, uses classification as a supervised learning approach to analyze datasets and create models for classifying data into desired classes [12].

Industry maintenance is crucial for equipment efficiency and operation. On the other hand, the preventive maintenance system is performed periodically, which can lead to increased costs and unexpected shutdowns. The basic principle of PdM is identifying touchable, quantifiable factors that indicate deterioration or aging [13]. The entities above are commonly referred to as reliability indicators. The surveillance of these indicators for differences or the prediction of their progression provides assessments of future malfunctions. The careful selection of appropriate indicators is of utmost importance, as the accuracy of calculations can only be deemed meaningful if the reliability indicators align with the actual physical condition of the machine. Identifying these signs necessitates a comprehensive understanding of the corresponding machinery. The enhancement or refinement of such methodologies is outside the scope of our methodology. Nevertheless, our efforts are directed towards integrating these technologies to acquire condition data automatically [14,15].

PdM uses predictive methodologies to ascertain the optimal timing for implementing maintenance interventions. The approach relies on the ongoing monitoring of the integrity of a machine or process, enabling maintenance activities to be carried out uniquely when necessary [15]. Furthermore, this technology allows for the timely identification of malfunctions by utilizing prediction algorithms that leverage historical data, such as machine learning techniques. Additionally, integrity considerations, including visual aspects, wear and tear, and deviations in coloration from the original, among other indicators, are taken into interpretation. Statistical inference methods and engineering approaches are also employed in this process.

PdM involves maintenance based on an estimate of a piece of equipment’s health status [16]. PdM is an advanced maintenance strategy that monitors equipment status to diagnose errors and estimate damage time. According to the research of Groba et al. [17], it involves defining status indicators, which are calculable parameters reflecting damage or the aging degree. The actual factory equipment status indicators are calculated using detected parameters. The state index modeling model is used to identify the dynamic characteristics of state indicators. The model predicts future trends of these indicators, and decision making is conducted by analyzing the prediction results and developing response strategies. This approach helps in reducing equipment damage and ensuring optimal equipment performance. Predictive technology is the fundamental component of the PdM plan and can be broadly categorized into three distinct approaches [18]. Several methods can be employed to assess errors from a statistical standpoint such as cluster analysis [19], statistical process control (SPC) [20], hidden Markov models (HMM) [21], and other statistical methods. Another method involves utilizing model-based approaches to develop a precise and well-defined mathematical model. While this approach can yield high accuracy, it also requires a deep program understanding. However, complex procedures often hinder the establishment of an accurate model.

The bidirectional nature of this contact has been facilitated by advances in artificial intelligence (AI) and big data, as well as the continuous enhancement of computer processing capacity [22]. The transformation above has not only been dominant in everyday life but has also been adopted in industrial and manufacturing settings regarding automation. To maintain a competitive edge, firms have increasingly relied on automation to enhance productivity [23]. The emergence of the Internet of Things (IoT) has been made possible by advancements in machine-to-machine (M2M) communication technologies. This notion permits interconnectivity and facilitates the active monitoring of systems within manufacturing appearances. The continuous transmission of data by these devices gives rise to the phenomenon known as big data, which necessitates a systematic approach to management within industrial settings. Big data enhances system performance and self-calibration, facilitating system coordination and feedback among various devices [24]. Predictive maintenance, using IoT and big data analytics, uses a predictive machine-learning model to predict equipment’s Remaining Useful Life based on historical data. The field of predictive maintenance is experiencing a surge in research efforts. Various types of equipment, such as bearings, turbofans, and engines, are the primary focus of these investigations. The primary objective is to identify the impending failure of such equipment by monitoring the intensity of vibrations. Predicting changes in vibration frequencies makes estimating the remaining operational lifespan possible. However, a significant challenge most researchers face in this domain is the limited availability of training data. This challenge becomes particularly pronounced when dealing with equipment with a longer service life, as obtaining sufficient damage data becomes difficult.

The proposal of the generative adversarial network (GAN) was put forth by Goodfellow et al. [25]. This framework involves the utilization of a generator and a discriminator to train an optimal generator that can generate synthetic data resembling accurate data. If this concept is applied to the domain of predictive maintenance, it has the potential to address the issue of insufficient experience with damages in real-world factory settings.

Lu et al. [26] introduced a novel approach that integrates generative adversarial networks (GANs) and LSTM models. This method effectively incorporates the training and prediction information of extended adversarial networks into the architecture of GANs. The proposed approach demonstrates improved capability in capturing the trend of deterioration curves.

This study aims to improve the Remaining Useful Life prediction and operational efficiency in industrial applications. The main challenge is to obtain sufficient for Remaining Useful Life prediction of MoSi_2_ heating elements in high-temperature environments. GANs network is applied to generate synthetic data resembling real-world damage. The main contribution to estimating the Remaining Useful Life of a MoSi_2_ heating element in a pusher kiln process lies in predictive maintenance strategies. By implementing a proactive approach that combines historical data analysis, real-time monitoring, and predictive modeling, organizations can anticipate potential failures and schedule replacements before they occur. This helps minimize downtime, reduce maintenance costs, and optimize the overall efficiency of the kiln process.

In this study, PdM architecture is applied to rotating bearing cases, further validating its effectiveness. It utilizes a specific architecture as a reference point and aims to implement it in the heating element of heating equipment within a natural factory setting. To achieve this, a substantial amount of data synthesized by a generative adversarial network is employed to train a regression model. Two prediction model selections by Long Short-Term Memory and Support Vector Regression models conduct a comparative analysis and identify the most appropriate machine-learning model for this particular case.

## 2. Process

**Figure 1:**
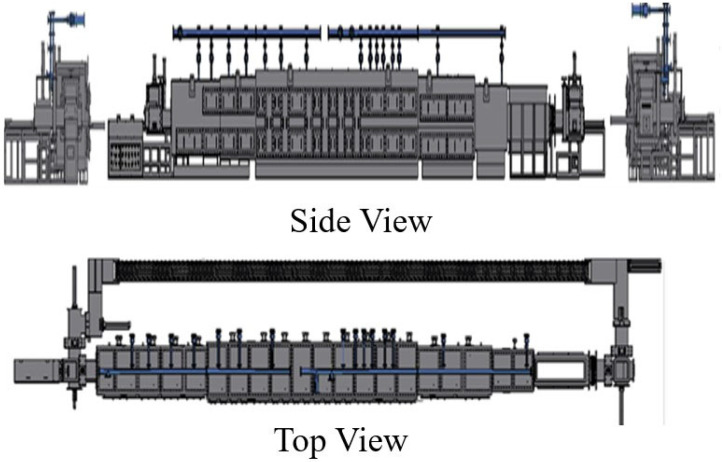
Push-plate sintering furnace (side and top view).

The push-plate sintering furnace is frequently utilized in the high-temperature sintering process of multilayer ceramic capacitors (MLCC). Within the sintering furnace, the ceramic chips undergo the necessary crystallization operation. The production line operates continuously, with approximately 50 plates constituting the circuit’s assembly. Each dish requires a minimum of 20 min for its movement process. Consequently, the duration for a single platen to complete the feeding and discharging is at least 1000 min. The sintering furnace has a total of 3 blocks and 15 heating zones (Heating Zones 1~6 in the heating block; Heating Zones 7~11 in the heating block; and Heating Zones 12~15 in the cooling block). Each heating zone is subdivided into Parts A and B, and different furnace areas of the heating furnace tube have 1~3 different pieces [27]. This research focuses on the heating section containing 10 heating areas (i.e., 7A; 7B; 8A; 8B; 9A; 9B; 10A; 10B; 11A; and 11B). The heating components in the heating zone are the subject of this research. The research data presented in this study, provided by Yageo Corporation Co., Ltd., (Kaohsiung, Taiwan) focus on collecting voltage, current, and temperature measurements in each heating zone. The sampling frequency for data collection is measured in days. A comprehensive dataset spanning three years has been accumulated. Figure 1 depicts the side and top views of the push-plate sintering furnace’s physical appearance.

### 2.1. MoSi_2_ Heating Element

Molybdenum disilicide, a ceramic metal, finds frequent application in high-temperature treatment and heating processes due to its possession of the following qualities [27]: molybdenum disilicide exhibits high-temperature resistance due to its ability to generate a protective oxide film in elevated temperature conditions. This film inhibits further oxidation and corrosion, enabling the material to function in extreme temperatures. Notably, molybdenum disilicide can operate at temperatures as high as 1700 °C [8], demonstrating its exceptional thermal stability and prolonged capacity for consistent heating performance under such conditions. The resistivity of molybdenum disilicide exhibits a pronounced increase as the ambient temperature rises. Consequently, when a fixed voltage is applied to the equipment, regions with lower ambient temperatures generate higher energy, resulting in rapid heating to the desired operating temperature. MoSi_2_ heating elements contribute significantly to the operational efficiency and thermal performance of pusher kilns by providing high-temperature capabilities, rapid and uniform heating, energy efficiency, durability, and oxidation resistance. Their versatility makes them a preferred choice for applications demanding precise and reliable high-temperature heating.

**Figure 2:**
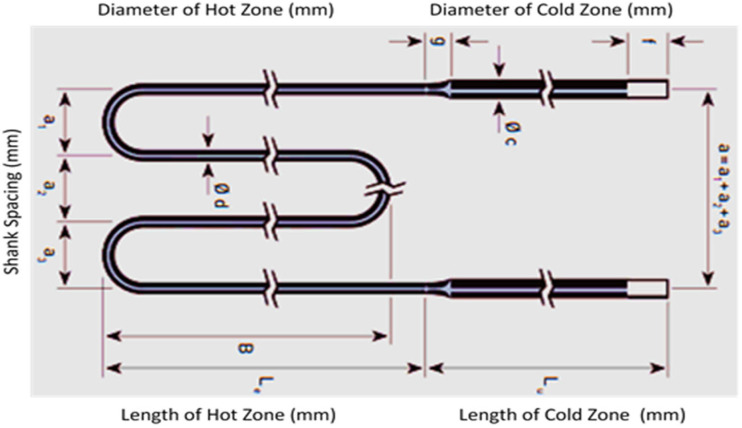
W-type molybdenum disilicate heating element.

The heating element exhibits a significantly elevated thermal conductivity, facilitating a uniform temperature distribution and effective thermal energy conversion, but it does not exhibit any signs of aging. The specifications of this model can be observed in Figure 2. The high-temperature end of the object has a W-shaped configuration, with a length (L_e_) of 400 mm and a diameter of 6 mm. On the other hand, the low-temperature end possesses a length (L_c_) of 560 mm and a diameter of 12 mm.

### 2.2. Maintenance

Maintenance is a growing field in multidisciplinary research, integrating data acquisition, infrastructure, storage, distribution, security, and intelligence. This section provides essential context for understanding maintenance and directs the study’s results. Maintenance costs account for 15–60% of manufacturing operating costs, but companies often mis-measure these costs. This review emphasizes the need for studies on new technologies to address this issue. The research focuses on maintenance growth and its role in Industry 4.0 implementation, highlighting its potential to be a significant factor in reducing maintenance costs [28].

The significance of high-temperature sintering in the manufacturing of Multi-Layer Ceramic Capacitors (MLCC) highlights the crucial role of maintaining consistent temperature during the process. The sintering furnace, operating at temperatures between 1100 °C and 1300 °C, utilizes nitrogen/hydrogen to prevent oxidation. The key challenge is ensuring uniform temperature across the furnace to promote the even and dense growth of the ceramic body’s crystal phase. The push-plate sintering furnace, equipped with multiple heating elements, requires consistent functioning for uninterrupted production. A failure in any heating element can lead to equipment shutdown, product defects, and substantial financial losses.

The study proposes the implementation of a maintenance strategy involving continuous monitoring of voltage, current, and other data through sensors. A machine-learning model is then employed to predict the deterioration of heating elements, estimate potential damage time, and attentive maintenance personnel for timely replacement. This approach aims to reduce unforeseen heating element failures and associated production disruptions. The predicted benefits of this study are threefold. Firstly, it aims to track equipment deterioration in real time, enabling the proactive replacement of components on the verge of failure. Predicting the Remaining Useful Life (RUL) of MoSi_2_ heating elements is crucial for implementing proactive maintenance strategies in industrial processes. This approach minimizes downtime, reduces costs, and optimizes resource allocation. It enhances safety in high-temperature environments, preserves product quality, and extends the lifespan of MoSi_2_ heating elements, contributing to long-term equipment performance and regulatory compliance. This approach ensures safer, more reliable, and cost-effective operation of industrial processes. This approach ensures continuous equipment operation, minimizing downtime and maintenance requirements, thereby enhancing productivity. Secondly, the study seeks to guarantee temperature consistency, leading to improved yield and reduced scrap losses from defective products. Thirdly, by eliminating unnecessary maintenance associated with regular schedules, the research aims to optimize the service life of heating elements and reduce overall waste. Maintenance strategies have evolved over industrial revolutions, with reactive maintenance being more common in understaffed manufacturing plants [29].

Predictive maintenance aims to prevent failures, reduce the risk of failure, and extend equipment life by intervening before failure occurs. Industries have struggled to abandon this maintenance philosophy due to its high costs and unplanned downtime. Adopting predictive maintenance in an industrial context is inevitable, but it faces challenges. Companies need to balance the benefits of predictive maintenance with the capital expenditure required to purchase necessary tools, software, and expertise. This disadvantage is particularly significant in the early stages of development when data on equipment behavior is uncommon, or new systems lack experience. This can lead to increased investment in predictive maintenance solutions. Challenges encountered in the scientific literature include financial and organizational limits, data source limits, machine repair activity limits, and deployment limits in industrial predictive maintenance models. While real-time monitoring is mentioned as a potential strategy, there may be a gap in how effectively this approach is integrated into existing maintenance practices. Implementing robust real-time monitoring systems that continuously collect data on key performance metrics, such as temperature profiles, electrical properties, and thermal efficiency, could enhance the accuracy of Remaining Useful Life estimates and enable proactive maintenance actions. Many existing predictive models for Remaining Useful Life estimation rely on simplified degradation models or empirical relationships, which may not capture the full complexity of degradation processes in pusher kiln environments. Developing more sophisticated predictive models, such as physics-based models or machine-learning algorithms trained on comprehensive datasets, could improve the accuracy and reliability of Remaining Useful Life predictions.

## 3. Predictive Maintenance

### 3.1. Machine Learning Algorithm

A machine learning (ML) model is a mathematical model that can be learned using machine-learning algorithms. Advanced monitoring infrastructures in modern power distribution systems enable fine-grained analytics and improved forecasting performance, particularly in electric load forecasting. Accurate load forecasting at the single-household level can create savings and reduce energy footprint [30]. Machine learning and statistical methods are used to train or calculate the probability of taking the maximum possible action in mahjong games, such as k-gate problems and search trees [31]. The study explored hybrid models for time series forecasting, following the positive results of single network-based models [32]. These algorithms apply historical data and prior experiences to determine the operational patterns that enable the model to make predictions, execute classifications, group data, or do other activities based on varying training data. The current research uses Python programming language and machine learning frameworks such as TensorFlow, Scikit-learn, and Keras to construct a modeling database. Machine learning models can be categorized into different types based on the availability of labels on training data, notable in Table 1. The excitation function enhances the expressive capacity of the neural network model by using non-linear equations, addressing its limitations, and tackling unsolvable problems. Without training the neural network with the excitation function, the output signal would be a basic linear function, resulting in a linear regression model. However, since real-world problems are inherently non-linear, the absence of non-linear functions would render the model meaningless and provide a summary of commonly perceived excitation functions [33].

### 3.2. Optimizer

While developing the ML model, the Optimizer is responsible for modifying the model’s parameters to minimize the loss function. Stochastic Gradient Descent (SGD) is treated as an optimizer for updating weight parameters in the gradient direction using differential techniques shown in Equations (1) and (2).

(1) $$V_{t}=\beta V_{t-1}-\eta \frac{\partial L}{\partial W}$$

(2) $$W_{t+1}=W_{t}+V_{t}$$

where *V_t_* represents the velocity of the direction. *L* represents the loss function, *W* denotes the weight parameter, and *η* signifies the learning rate. This technique enhances the learning process by accelerating the learning rate. If *V_t_* aligns with the previous update direction, a larger *V_t_* will result in a higher gradient for the weight update. Conversely, a smaller *V_t_* will lead to a decrease in the rise. The drag coefficient, denoted as *β*, is typically assigned a value of 0.9.

### 3.3. Generative Adversarial Networks

The advantage of Generative Adversarial Networks (GANs) is that they provide a framework for learning the underlying probability distribution of a dataset, allowing for the generation of new samples that are indistinguishable from real data. However, the disadvantages of GANs are notoriously unstable and sensitive to hyperparameters, often requiring careful tuning and experimentation to achieve good results. Mode collapse, vanishing gradients, and oscillations in training dynamics are common challenges that can hinder the convergence [34]. Goodfellow et al. [25] introduced the Generative Adversarial Network (GAN) structure, which trains two opposing neural networks: a generator, G, and a discriminator, D. The generator generates synthetic data like real data. In contrast, the discriminator distinguishes between real and synthetic data. The competition is formulated into a two-player minimax game defined by the value function *V*(*G*, *D*), improving the generated data quality until the discriminator fails. The minimax game is given by Equation (3).

(3) $$\underset{G}{\min}\underset{D}{\max}V\left(G,D\right)=E_{x\sim p_{data}\left(x\right)}\left[logD\left(x\right)\right]+E_{x\sim p_{z}\left(z\right)}\left[log\left(1-D\left(G\left(z\right)\right)\right)\right]$$

where *x* is the real data, *z* is the random noise, *D*(*x*) denotes the output given by the discriminator when the input is *x*, and *G*(*z*) denotes the generated data.

**Figure 3:**
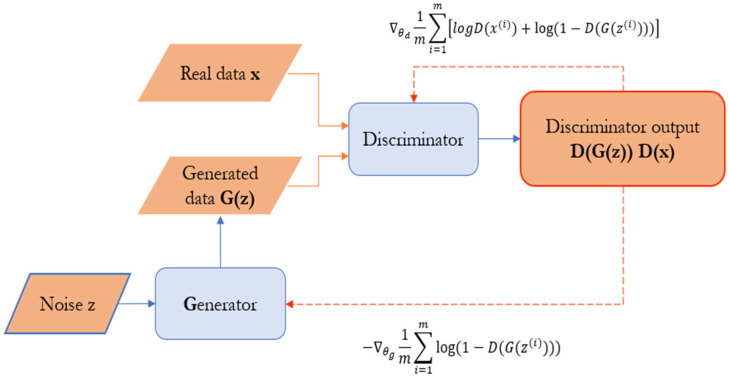
Generative adversarial network architecture.

The basic architecture of the GAN model is shown in Figure 3 [35]. It aims to develop a generator to provide the highest fidelity synthetic data. The study uses a Generative Adversarial Network (GAN) to generate simulated failure scenarios in situations where real-life failure experience is limited. GANs are artificial intelligence models that work together to create synthetic data, with the generator creating data and the discriminator evaluating its authenticity. This approach allows for the generation of realistic failure patterns, mimicking the behavior of MoSi_2_ heating elements as they degrade over time. This synthetic data generation improves the robustness of predictive models and allows for more accurate estimation of the Remaining Useful Life (RUL) in scenarios without extensive real-world failure data. GANs are a valuable tool for addressing data scarcity challenges and enhancing prediction reliability in scenarios with limited actual failure events. The execution of the objective function involves periodically altering the parameters in a back-and-forth method. The steps for this procedure are as follows: (i) the generator G parameters are held constant while modifying the parameters of the discriminator D to maximize the discrimination degree *V*(*G*, *D*), resulting in the acquisition of the optimal discriminator; (ii) optimize the discriminator while altering the parameters of the generator G to minimize the recognition; (iii) iterate steps (i) and (ii) to generate synthetic data that closely resembles the actual data.

### 3.4. Regression Models

The Long Short-Term Memory (LSTM) network is well suited for processing significant events with long intervals and delays in time series data. The input value undergoes processing through three control gates (gates) to determine the storage and utilization of information. These control gates, namely the forgetting gate, input gate, and output gate, regulate whether the information modifies the cell state (cell state) and whether the cell state continues to be propagated [26]. The forget gate operation determines how much information carried over from the previous layer’s output will be disregarded and not propagated to the subsequent layer. The forget gate serves the purpose of deciding whether the prior information should be discarded or preserved.

The Support Vector Regression (SVR) model is founded upon expanding the Support Vector Machine (SVM) method designed for regression analysis. The model initially identifies a hyperplane in the high-dimensional space, then it constructs a pipeline around the hyperplane with a margin of ε on both sides. The loss function is not computed for all samples within the pipeline. Instead, the error arises from support vectors outside the pipeline. SVR aims to find a hyperplane that fits data with minimal error, like linear regression models. The SVR optimization algorithm is required to minimize the tube’s size.

### 3.5. Methodology

**Figure 4:**
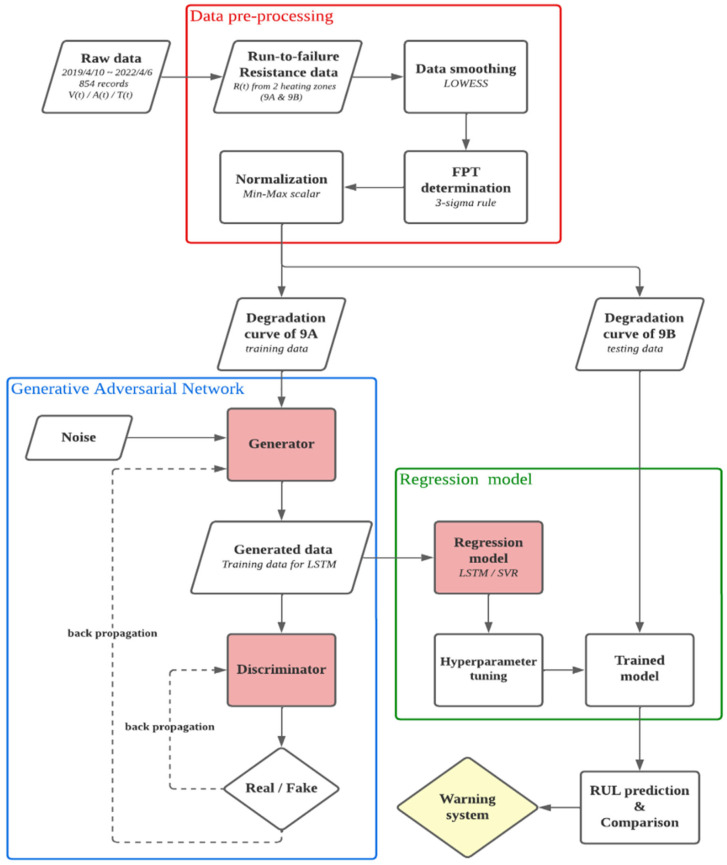
Flowchart of the proposed machine-learning algorithm.

The flowchart of the proposed machine-learning algorithm is shown in Figure 4. The furnace equipment consists of a total of 15 furnace areas, each of which is further divided into two parts, namely A and B. As the primary focus of the heating zone, each furnace zone is outfitted with three sensors that gather data on voltage, current, and temperature. The sampling frequency is set at 1 reading every 30 s. These readings are stored in the SD card of the acquisition device until a sufficient amount of data is accumulated. At that point, the data are manually transferred to a computer for storage. Given the minimal daily fluctuations in data, the original data are documented on a daily basis. The equipment data have been accumulated over approximately three years, specifically from 18 June 2020, to 7 April 2022. During this period, instances of furnace tube breakage and subsequent replacements have been meticulously recorded. To create a machine-learning model capable of forecasting the deterioration curve of a heating furnace tube, it is necessary to get historical data about past instances of degradation. However, since heating furnace tubes typically last for 4 to 5 years, it is challenging to collect data on the tubes from replacement to rupture period. The raw data only record two unexpected fractures and their occurrence times.

The heating method of the molybdenum disilicide heating element is based on resistance heating. It uses current to generate resistance heat, which is then transferred to the environment as heat energy. Over time, the surface of the heating element gradually peels off, causing the diameter of the heating section to decrease and the resistance to increase. Eventually, when the resistance reaches infinity, the heating element breaks. By monitoring the change in resistance, we can assess the health of the heating element. In this study, the resistance value is used as an indicator of the heating element’s condition. The simplest method of creating a virtual health index is by transforming raw sensor data using mathematical functions. In most cases, a virtual health index is built using physical sensor measurements with one or multiple objectives, such as improving the correlation between the health index and the RUL, reducing the signal noise, and removing nonmonotonic behavior. Remaining Useful Life (RUL) is a crucial concept in industrial equipment operation, guiding proactive maintenance and resource optimization. It allows organizations to schedule maintenance activities, minimize downtime, and optimize resource allocation. This proactive approach enhances operational efficiency, reduces costs, and extends equipment longevity. Predicting RUL helps in ensuring safety and compliance with regulatory standards. In industrial applications, RUL is measured using historical data, sensor readings, and predictive models, using techniques like condition monitoring and machine-learning algorithms. The goal is to balance equipment lifespan with the risk of unexpected failures, ensuring reliable and efficient industrial system operation.

To create a deterioration curve, data smoothing is performed on the original data to remove noise and volatility, highlighting trends and allowing for analysis of the data. Exponential smoothing is a smoothing method based on a weighted average, using a smoothing coefficient α (0 < α < 1). The smoothed value of the previous data and the actual value of the current data are weighted and averaged to calculate the smoothing value of the current data, which is suitable for data with trend and seasonality. Locally Weighted Scatterplot Smoothing (LOWESS) fits the polynomial regression curve in a specific regional window and weights the distance between each point of the regional window and the current data point, which is conducive to observing the data in the region. The larger the proportion of the regional window, the smoother the data, but with the relative sacrifice of fitting accuracy.

First Prediction Time (FPT) and End of Life (EOL) are the start and end points of the deterioration curve, respectively. Two common methods of data regularization are Min–Max Normalization and Rewrite–Minimize Normalization. Min–Max Normalization scales data to 0-1, requiring redefining the maximum and minimum values. However, this method has a disadvantage as processed data do not have zero meaning, suggesting the data center is not at the origin. The molybdenum disilicide heating element is a resistive heating method that uses current to heat the heating element, transferring heat energy to the environment. As the heating element’s surface peels off over time, its resistance increases until it breaks when it reaches infinity.

This study uses the resistance value as the state index of the heating element, capturing the two heating zones (9A/9B) over a year before equipment damage. The change in resistance is observed to reflect the current health of the heating element. There are only two sets of real damage data, one of which (9A) will be used as training data to generate a large amount of synthetic data similar to training data in the adversarial network, and the other (9B) will be used as test data because the test data must be new information that the model has not seen before it can be evaluated with the reference value. In this case, failure experience only happens twice, which is in Heating Zones 9A and 9B. A Generative Adversarial Network (GAN) structure that simultaneously trains two opposing neural networks: a generator and a discriminator. The generator G takes in random noise z and tries to generate synthetic data that are similar to the real data. The discriminator D tries to distinguish between real data and synthetic data. Therefore, GAN is trained on data from 9A to generate sufficient training data for the LSTM model. Then, data from 9B are used as testing data to evaluate the performance of the LSTM model. To show the quality of the forecasting model, testing data must be assumed to be future or unknown data, so it cannot participate in the generation of training data.

The goal of SVR is to find a hyperplane that can fit the data with a minimum error, like a linear regression model. The difference is that it introduces an ε-insensitive tube where loss is neglected for data included. The slack variable is also introduced for some data outside the tube. After regression models are tuned, they are tested by both curves. However, only Degradation Curve 9B has a reference value since it is unknown data for both models. MAE, RMSE, and R-squared are used as evaluation metrics. The Remaining Useful Life (RUL) using deep-learning techniques for predictive maintenance is based on three different data types: lifetime data, run-to-failure data, and threshold data. The final step is to set a pre-defined threshold so that a warning would be triggered when the resistance value becomes abnormal. In this case, a warning would be triggered if resistance exceeds 10% to 20% of the normal value. Machine-learning models predict Remaining Useful Life (RUL) using historical data, including sensor readings and operational parameters. These data are analyzed through feature engineering to identify health and degradation patterns. The model is trained on the training set, followed by validation and hyperparameter tuning. The richness and diversity of historical data contribute to its effectiveness in accurately predicting equipment’s RUL.

## 4. Results and Discussion

The data used in this institute comes from the sintering furnace of passive component used by Guogeo Corporation Co., Ltd. located in the Nanzi plant area of Kaohsiung. The equipment has a total of 15 furnace areas. The furnace area is divided into two parts: Parts A and B, with heating areas ranging from 7 to 11 and cooling areas from 13 to 15. A predictive maintenance strategy is primarily used for the heating furnace tube in the heating zone. Each furnace zone in the heating zone is equipped with three sensors, which collect voltage, current, and temperature, respectively, and the sampling frequency is 1 every 30 s, which is stored in the SD card of the acquisition device until a certain amount of data are collected and then manually stored in the computer. Since the daily data change very little, the original data are recorded in days. The equipment data have been collected for about three years (18 June 2020~7 April 2022), and the time of the furnace tube damage and replacement has been recorded.

To establish a machine-learning model that can predict the deterioration curve of the heating furnace tube, the past deterioration experience must be used as training data for the model to learn, but due to the long service life of the heating furnace tube, an average of about 4~5 years, it is quite difficult to completely obtain the data of the heating furnace tube from the replacement to the fracture period, and the existing raw data only records two fractures without warning, and its occurrence time and location. Data pre-processing consists of the following four steps: defining status indicators, data smoothing, plotting deterioration curves, and data regularization are explained in detail in Supplementary Material (Figures S1–S4).

**Figure 5:**
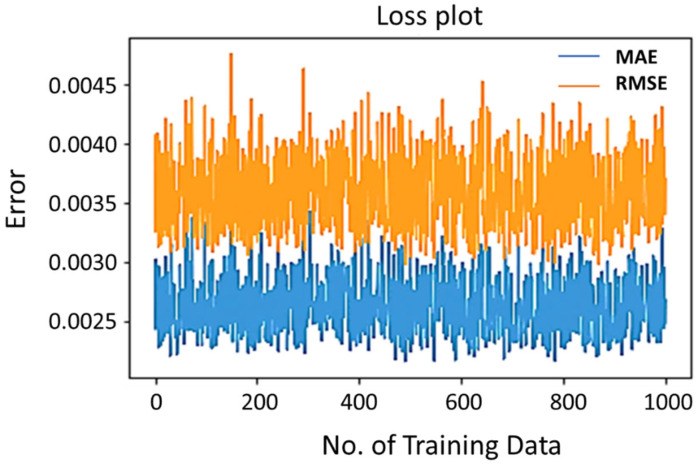
Influence of training data on SV.

**Figure 6:**
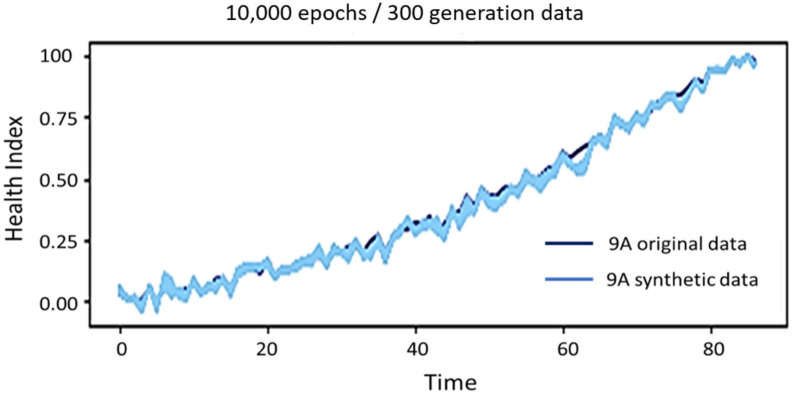
Synthetic data for generating adversarial networks.

In the current study, data are assembled regularly from 10 April 2019 to 6 April 2022. During this particular time frame, instances of failure were observed in two specific heating zones, 9A and 9B. Consequently, the Generative Adversarial Network (GAN) is trained using data from 9A to produce ample training data for the Long Short-Term Memory (LSTM) model. Subsequently, the data obtained from 9B are utilized as testing data to assess the LSTM model’s efficacy. To evaluate the effectiveness of a forecasting model, it is imperative to designate the testing data as future or unknown data, excluding its involvement in developing the training data shown in Figure 5. The degradation curve of 9A is initially inputted into a Generative Adversarial Network (GAN) to provide a substantial quantity of training data. Given the complex nature of the problem and the evaluation metric of Long Short-Term Memory (LSTM) and Support Vector Regression (SVR) models concerning the impact of the training data size on model performance, we have decided to employ 10,000 epochs for training the Generative Adversarial Network (GAN). Consequently, we will build a dataset of 300 instances to train the regression model shown in Figure 6.

The evaluation of synthetic data quality often relies on commonly used metrics such as Root Mean Square Error (RMSE), Mean Absolute Error (MAE), and Percent Root Mean Square Deviation (PRD), which are shown in Equations (4)–(6).

(4) $$RMSE=\sqrt{\frac{1}{N}\sum_{i=1}^{N}{\left(O_{i}-S_{i}\right)}^{2}}$$

(5) $$MAE=\frac{1}{N}\sum_{i=1}^{N}\left|O_{i}-S_{i}\right|$$

(6) $$PRD=\sqrt{\frac{\sum_{i=1}^{N}{\left(O_{i}-S_{i}\right)}^{2}}{\sum_{i=1}^{N}{O_{i}}^{2}}}*100\%$$

The effectiveness of a Remaining Useful Life (RUL) prediction model is evaluated using metrics like Mean Absolute Error (MAE), Root Mean Squared Error (RMSE), and R-squared values. These metrics measure the model’s accuracy in predicting actual RUL values, with a lower MAE and RMSE indicating better performance. R-squared values measure the proportion of variance in the RUL. The model’s generalizability to new data is also crucial. Long Short-Term Memory (LSTM) and Support Vector Regression (SVR) models are compared for RUL prediction, considering factors like their predictive accuracy, robustness, and computational efficiency. Furthermore, the quantity of synthetic data is contingent upon the intricacy of the case and lacks a predetermined requirement. Consequently, this study employs various training data sizes to train the model and determine the optimal training data based on the model’s prediction error and time consumption. The changes in error for the two regression models use training data ranging from 0 to 1000. The extended short-term memory network demonstrates exceptional predictive performance with only approximately 300 training data instances. Despite the Support Vector Regression model exhibiting a relatively low deviation, it consistently experiences oscillation within a specific range that cannot be reduced. This study addresses this issue by uniformly training the model using a dataset of 300 instances. Additionally, it is observed that increasing the number of iterations (epoch) leads to improved synthesis outcomes. However, this improvement comes at the cost of increased computational energy and time consumption. After careful consideration, a compromise is made, and the model is trained for 10,000 iterations. The design of the generative adversarial network is based on the architecture used in the study by Lu et al. [26], as shown in Table 2.

The generative adversarial network generates synthetic data that accurately reflect real data trends, demonstrating low error between real and synthetic data, requiring no model architecture modification. The study utilizes two LSTM layers and a fully connected layer in a Long Short-Term Memory network model, adjusting hyperparameters like time lag, dropout rate, and the number of neurons. The study indicates that the optimal hyperparameter combination can achieve a RMSE of 0.0031. The Long Short-Term Memory network model uses two LSTM layers and one fully connected layer for its hidden layer. Hyperparameters like the number of neurons and dropout rate are adjusted using a grid search shown in Table 3. The model makes predictions based on past time lag and feature length. The best combination of hyperparameters is achieved when the hidden layer has 256 neurons and a 0.25 discard rate, achieving a RMSE of 0.0049. Regression models are tested using both curves, with Degradation Curve 9B as a reference. MAE, RMSE, and R-squared metrics are used, with prediction results shown in Equation (7).

(7) $$R^{2}=1-\frac{\sum_{i=0}^{m}{\left(y_{i}-{\hat{y}}_{i}\right)}^{2}}{\sum_{i=0}^{m}{\left(y_{i}-{\bar{y}}_{i}\right)}^{2}}$$

Support Vector Regression (SVR) is a machine-learning algorithm that accurately predicts resistance variation in predictive modeling tasks, especially when dealing with non-linear relationships in data. It uses support vectors and a kernel function to map input features into a higher-dimensional space, capturing complex patterns and non-linear relationships. The R-squared (R2) value is used to assess the level of prediction accuracy, which represents the proportion of variance in the dependent variable explained by the model. A higher R2 value indicates a better fit to the actual data, with a maximum of 1.0 indicating a perfect fit.

**Figure 7:**
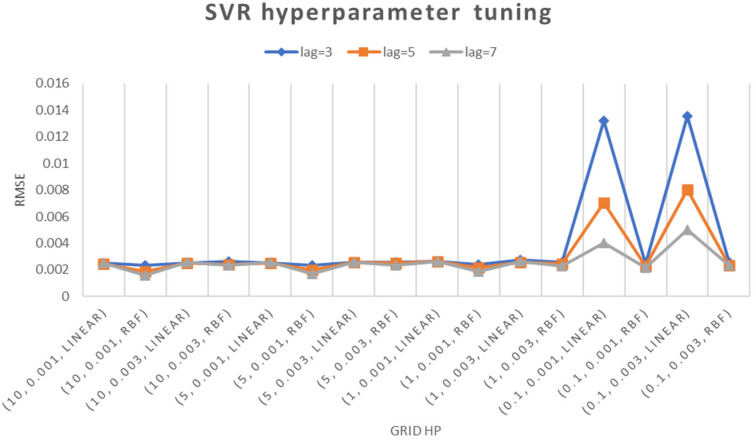
Hyperparameter adjustment of Support Vector Regression model.

SVR is a crucial tool in industrial operations for predicting equipment safety. It accurately predicts the Remaining Useful Life of critical components, reducing the risk of unexpected breakdowns. SVR’s predictive maintenance helps in reducing unplanned downtime and enhancing overall safety. It also contributes to equipment reliability and compliance with safety regulations, fostering a safer working environment. Integrating SVR into real-time monitoring systems allows for continuous updates on equipment health, enhancing safety measures and promoting the overall safety and reliability of industrial processes. Overall, SVR significantly enhances safety measures and reduces risks. The grid search method is applied to SVR, adjusting four hyperparameters: time lag, kernel function, cost, and epsilon, resulting in a RMSE of 0.0016 using the best hyperparameter combination. The grid search results are compared in Figure 7, and the best combination of hyperparameters is shown in Table 4.

**Figure 8:**
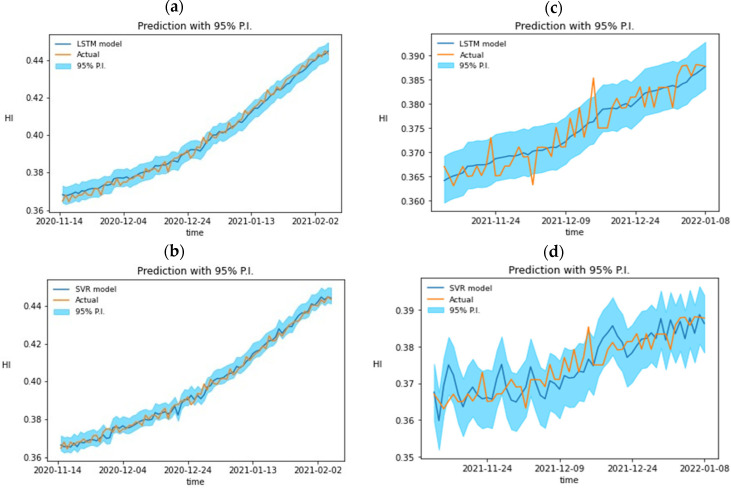
Prediction curves of HI vs. time for 9A and 9B heating elements by using (**a**) LSTM, (**b**) SVR, and (**c**) LSTM, (**d**) SVR, respectively.

Since the test data do not participate in the training of the adversarial network and the regression model, the data can be identified as unknown data to ensure that the model can maintain its accuracy when predicting future data, and the mean absolute error (MAE), root mean square error (RMSE) and R-squared (R-squared) are selected as indicators to evaluate the predictive ability of the model. Figure 8a,b is the prediction results of LSTM and SVR for 9A, while Figure 8c,d is the prediction results of LSTM and SVR for 9B to use the residual shoe-pulling method for obtaining 95% of the prediction interval.

Evaluation metrics include MAE, RMSE, and R-squared. The prediction performance of the regression model is shown in Table 5, and the advantages and disadvantages of the two regression models can be compared with the histogram. Based on the results of unknown data 9B, the MAE of both models tends to be close to 0, indicating that the average prediction ability of the two models is very strong. However, the comparison results of RMSE and R^2^ show that the predicted value of SVR can better reflect the actual resistance change.

### Warning System

Predictive maintenance uses machine-learning models to predict equipment damage and find the best maintenance time. This study uses resistance values to assess the health of molybdenum disilicide heating elements. When resistance exceeds 10% of normal, warnings are issued. When resistance exceeds 20%, warnings are issued.

**Figure 9:**
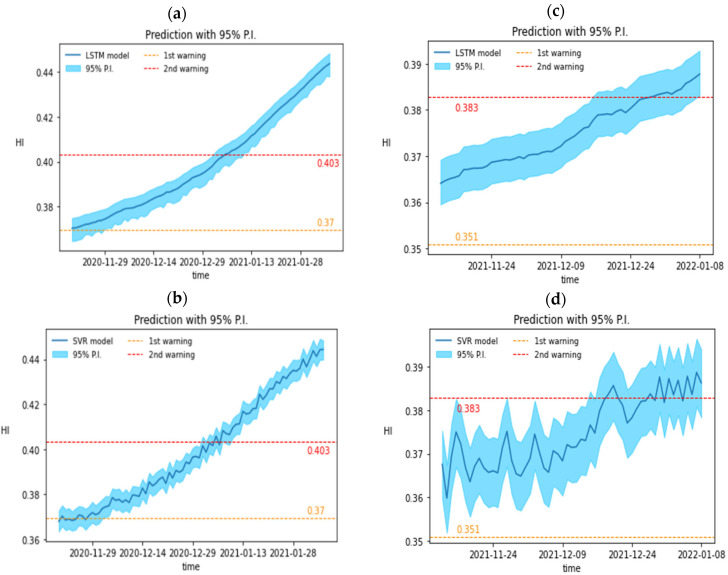
Prediction of the RUL of 9A and 9B heating elements by using (**a**) LSTM, (**b**) SVR, and (**c**) LSTM, (**d**) SVR, respectively.

The outcomes of the warning system are coupled with the probabilistic prediction of heating elements in the heating zone of 9A and 9B by LSTM. The outcomes of the SVR probabilistic prediction are in conjunction with the warning system for the heating elements in the respective heating zones 9A and 9B. The two warning levels, the actual damage amount, and the day that the anticipated resistance of the LSTM and SVR will, respectively, cause the alert to be triggered are all noted. The second warning was issued 20 days before the damage date and 10 days before the LSTM due to severe resistance oscillations in the predicted SVR resistance. Figure 9a,c is the results of LSTM’s probabilistic prediction of heating elements in the heating zone of 9A and 9B combined with the warning system. Figure 9b,d is the results of SVR probabilistic prediction combined with a warning system for heating components in the 9A and 9B heating zones, respectively. Table 6 records the two warning thresholds, the actual damage value, and the date when the predicted resistance of LSTM and SVR will trigger the warning, respectively. Based on the results of unknown data 9B, the second warning was triggered earlier, about 20 days from the actual damage date and about 10 days from the LSTM, due to the severe oscillation of the predicted resistance of the SVR.

## 5. Conclusions

This study establishes that estimating the Remaining Useful Life (RUL) of MoSi_2_ heating elements in a pusher kiln process is a multifaceted task influenced by various factors. The operating temperature, thermal cycling, atmospheric conditions, mechanical stress, maintenance practices, element quality, and adherence to manufacturer recommendations all play pivotal roles in determining the longevity of these heating elements. Two regression models, namely Long Short-Term Memory (LSTM) and Support Vector Regression (SVR), are compared, and their hyperparameters are adjusted using grid search to enhance model performance. Additionally, a generative adversarial network generates substantial synthetic data for training the regression model. Nevertheless, when examining the root mean square error (RMSE) and coefficient of determination (R^2^), it becomes evident that Support Vector Regression (SVR) is more adept at capturing the variations in actual resistance. The R^2^ value can be 0.634, indicating a superior level of fitting accuracy. Moreover, the study recommends ongoing research to refine and validate the RUL prediction models, considering variations in operational parameters and exploring potential mitigation strategies. The outcomes of this investigation contribute to the broader understanding of high-temperature material degradation in industrial settings and provide a foundation for proactive maintenance practices in pusher kiln processes employing MoSi2 heating elements. However, it is important to acknowledge the need for a nuanced approach, considering the unique circumstances of each specific application. Collaborating with manufacturers and specialists in industrial heating systems is advisable to obtain accurate estimations tailored to the specific operational conditions. Consequently, two threshold values, Warning 1 and Warning 2, are developed for optimal maintenance intervals when the usual resistance value increases by 10% and 20%, respectively.

The predictive resistance of Support Vector Regression (SVR) exhibits a higher degree of realism. However, it is important to note that SVR’s huge vibration amplitude has a higher likelihood of activating the warning system, and this activation occurs approximately 10 days before the occurrence predicted by Long Short-Term Memory (LSTM). About safety considerations, Support Vector Regression (SVR) demonstrates a notable level of predictive accuracy. Even though it may prompt an earlier warning, it allows ample time to establish maintenance plans. Consequently, SVR is a more appropriate choice for the predictive maintenance technique employed in this study.

In future research endeavors, it is imperative to delve deeper into the estimation of the RUL of MoSi_2_ heating elements in pusher kiln processes. Advanced monitoring techniques, such as real-time sensor data analysis, and the development of predictive maintenance models using machine-learning algorithms offer promising avenues for refining RUL predictions. Additionally, exploring material improvements, optimizing operating parameters, and fostering collaborative research efforts among industry stakeholders can collectively contribute to enhancing the durability and efficiency of MoSi_2_ heating elements. Case studies documenting the performance of these elements under varying conditions and participation in standardization efforts further ensure a comprehensive and standardized approach to managing these crucial components in industrial heating applications. Addressing these future considerations will be instrumental in advancing our understanding and implementation of effective maintenance strategies for MoSi_2_ heating elements in pusher kiln processes. Although previous studies have observed that deep-learning techniques (e.g., Generative Adversarial Networks, Recurrent Neural Networks, Deep Belief Networks, Convolutional Neural Networks, etc.) attract growing interest in RUL prediction and suggests a promising future for their applications in manufacturing, no study focused on the RUL prediction of MoSi2 heating elements [36].

## Figures and Tables

**Table 1:** Common excitation functions.

Function Name	Noted	
Second, the focus curve function (Sigmoid function)	graphics	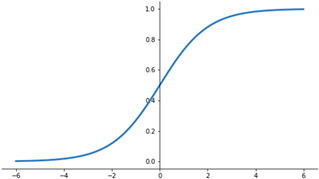
	equation	$\sigma (x)=\frac{1}{1+e^{-x}}$
Hyperbolic tangent function (Hyperbolic tangent function, tanh)	graphics	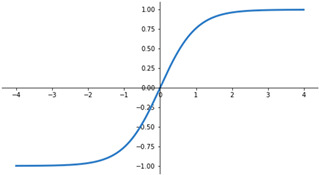
	equation	$tanh(x)=\frac{e^{x}-e^{-x}}{e^{-x}+e^{x}}$
Rectified linear function (Rectified Linear Unit, ReLU)	graphics	
	equation	R(x)=max(0, x)
Leakage rectification linear function (Leaky ReLU)	graphics	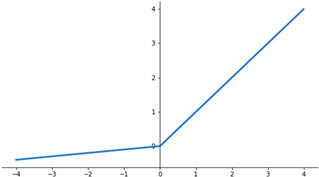
	equation	LR(x)=max(0.1x, x)

**Table 2:** Generation adversarial network architecture.

Module Name	Layer	Parameter
Generator	Fully connected layer	Output shape = (sample, 128) Activation = LeakyReLU
	Fully connected layer	Output shape = (sample, 128) Activation = LeakyReLU
	Fully connected layer	Output shape = (sample, n_output) Activation = tanh
Discriminator	Fully connected layer	Output shape = (sample, 64) Activation = LeakyReLU
	Fully connected layer	Output shape = (sample, 128) Activation = LeakyReLU
	Fully connected layer	Output shape = (sample, 64) Activation = LeakyReLU
	Fully connected layer	Output shape = (sample, 1) Activation = Sigmoid

**Table 3:** LSTM grid search result.

	Grid Parameter	Best Parameter	RMSE
Time lag	[3, 5, 7]	7	0.0031
Number of neurons	[64, 128, 256]	256	
Dropout rate	[0.2, 0.25]	0.25	

**Table 4:** SVR grid search result.

	Grid Parameter	Best Parameter	RMSE
Time lag	[3, 5, 7]	7	0.0016
Kernel function	[Linear, RBF]	RBF	
C	[10, 5, 1, 0.1]	10	
ε	[0.001, 0.003]	0.001	

**Table 5:** Prediction performance of the regression model.

		MAE Test	RMSE Test	R^2^ Test
LSTM	9A	1.21×10^−5^	0.00348	0.97864
	9B	3.21×10^−5^	0.00567	0.45889
SVR	9A	4.16×10^−6^	0.00204	0.99268
	9B	2.17×10^−5^	0.00466	0.63352

**Table 6:** Heating element warning threshold and date.

Threshold						
	Normal Resistance Value	Warning1		Warning 2		Actual damage value
9A	0.336	0.369		0.403		0.443
9B	0.319	0.351		0.383		0.387
			Warning 1 date		Warning 2 date	
9A	Actual		12-11-2020		06-01-2021	
	LSTM		12-11-2020		29-12-2020	
	SVR		13-11-2020		29-12-2020	
9B	Actual		/		02-01-2022	
	LSTM		/		22-12-2022	
	SVR		/		12-12-2021	

## Data Availability

Data are contained within the article.

## Supplementary Materials

The following supporting information can be downloaded at: https://www.mdpi.com/article/10.3390/s24051486/s1.

## Nomenclature

AL	Artificial Intelligence
CPS	Cyber–Physical System
EOL	End of Life
FPT	First Prediction Time
GANs	Generative Adversarial Network
HMM	Hidden Markov Model
LOWESS	Locally Weighted Scatterplot Smoothing
LSTM	Long Short-Term Memory
IoT	Internet of Things
MAE	Mean Absolute Error
ML	Machine Learning
MLCC	Multilayer Ceramic Capacitors
MoSi_2_	Molybdenum disilicate
M2M	Machine-To-Machine
PdM	Predictive Maintenance
PRD	Percent Root Mean Square Deviation
RMSE	Root Mean Square Error
RUL	Remaining Useful Life
SGD	Stochastic Gradient Descent
SPC	Statistical Process Control
SVM	Support Vector Machine
SVR	Support Vector Regression
