# Supplementary material for: Remaining Useful Life Estimation of MoSi2 Heating Element in a Pusher Kiln Process
Source: Sensors (Basel). 2024 Feb 24;24(5):1486. doi: 10.3390/s24051486 (PMC10933939; doi:10.3390/s24051486)
Supplement: Supplementary file 1 [file sensors-24-01486-s001.zip › sensors-2845073-supplementary.pdf]

1. Health Indicator

The molybdenum disilicide heating element heating method belongs to resistive heating, the use of current through the generated resistance heat to heat up, thereby transferring heat energy to the environment; with the increase of use time, the surface of the heating element will slowly peel off so that the diameter of the heating section is getting smaller and smaller, the resistance also increases, until the heating element breaks when the resistance reaches infinity, by observing the change of resistance, can reflect the current health of the heating element. Therefore, this study uses the resistance value as the state index of the heating element, Figure S1, to capture the two heating zones(9A/9B). The change in resistance over the period of one year prior to the damage of the equipment.

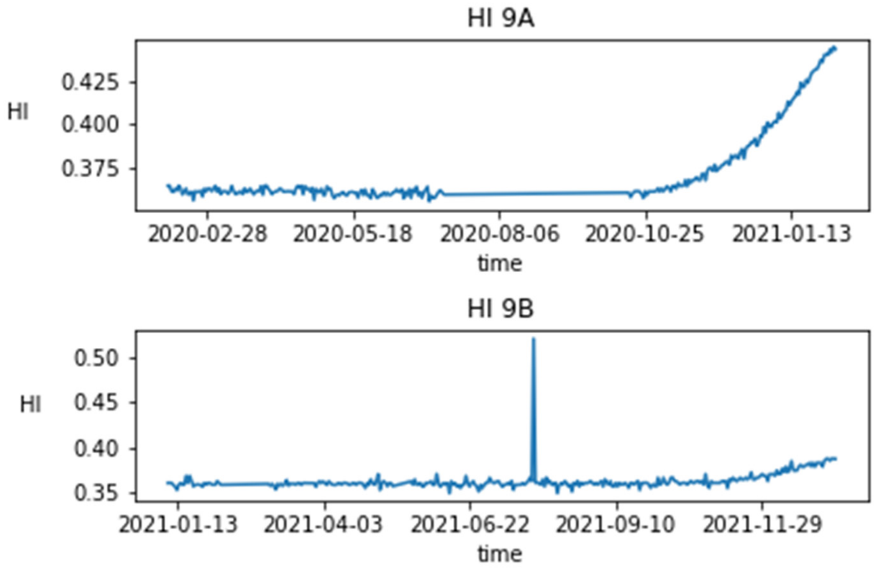

Figure S1. Change of status indicators (9A/9B).

2. Data Smoothing

Before drawing the deterioration curve, it is necessary to do data smoothing processing on the original data, the purpose is to highlight the trend of the data by removing noise and volatility, and also make the analysis of the data not affected by outliers, there are three common data smoothing methods [15] moving average (moving average) uses equation (1), to calculate the average value in a specific window around the data point to replace the data point, and the window size determines the

smoothness of the data; Exponential smoothing is a smoothing method based on weighted average, such as equation (2), using a smoothing coefficient α (0<α<1) The smoothed value of the previous data and the actual value of the current data are weighted averaged to calculate the smoothing value of the current data, which is suitable for data with trend and seasonality; Locally Weighted Scatterplot Smoothing (LOWESS) fits the polynomial regression curve in a specific regional window, as shown in equations (3) and (4) and weights the distance between each point of the regional window and the current data point according to equation (5), which is conducive to observing the data in the region the larger the proportion of the regional window, the smoother the data, but the relative sacrifice of fitting accuracy. Since LOWESS is a local weighted regression model and is more flexible in eliminating outliers, this study expects to eliminate the influence of outliers on the interpretive data of the machine learning model through this smoothing method, and the smoothed state indicators are shown in Figure S2.

$$S_t = (y_{t-k} + y_{t-k+1} + y_{t-k+2} + \dots + y_t)/k \quad (1)$$

$$S_t = \alpha * y_t + (1 - \alpha) * S_{t-1} \quad (2)$$

$$S_t = \hat{\beta} X_i, \ where\ X_i = \begin{bmatrix} 1 & x_1 \\ \vdots & \vdots \\ 1 & x_N \end{bmatrix} \quad (3)$$

$$\hat{\beta} = (X^T W X)^{-1} X^T W_y, \ where\ W = \begin{bmatrix} w_1 & \cdots & 0 \\ \vdots & \ddots & \vdots \\ 0 & \cdots & w_N \end{bmatrix} \quad (4)$$

$$w_i = \left(1 - |d_{ij}|^3\right)^3 \quad (5)$$

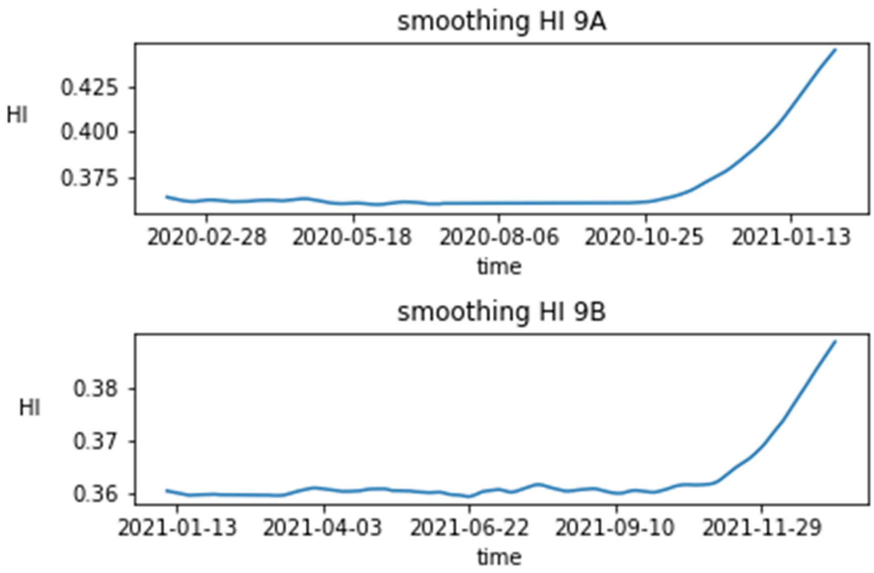

Figure S2. Smoothing state index (9A/9B).

3. Degradation curve

First Prediction Time (FPT) and End of Life (EOL) are the start and end points of the deterioration curve, respectively. Under normal circumstances, the sensor will continuously monitor the state of the equipment and calculate the mean and standard deviation of the normal data, when there are two consecutive data points beyond the three standard deviations of the mean, the model is driven and predicts the data, this time point is the starting prediction time, when the model's predicted value exceeds the pre-defined threshold value to stop predicting, this time point is the equipment termination time, and the state change between these two points is the deterioration curve of the equipment are shown in Figure S3 [16].

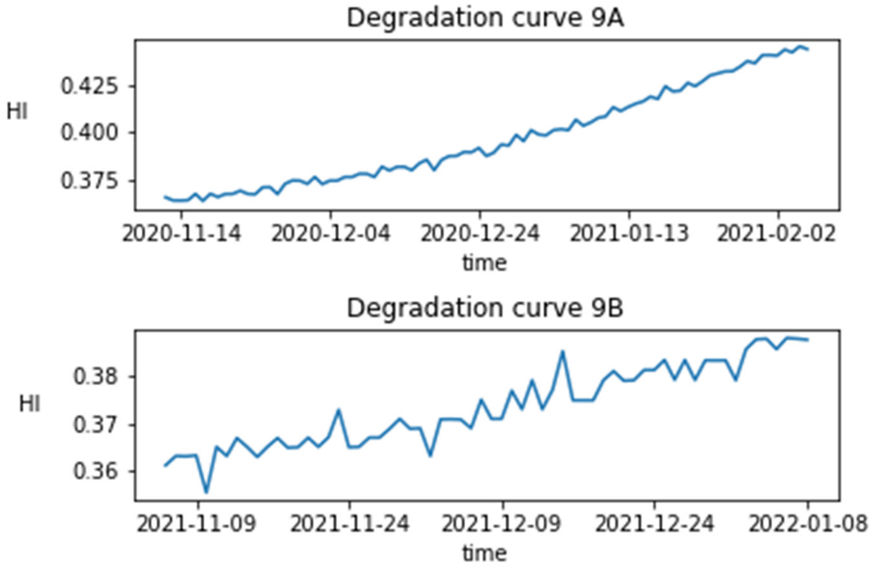

Figure S3. Deterioration curve (9A/9B).

4. Data Normalization

There are two common ways of data regularization: Min-Max Normalization uses equation (6) to scale the data to the interval of 0 ~ 1, if the original data has new data added, the maximum and minimum values in the data in the formula must be redefined, the disadvantage of this method is that the processed data does not have zero meaning, meaning that the data center is not at the origin; Mean normalization (Mean Normalization) by subtracting the mean to shift the data to the interval of -1 ~ 1, see equation (7), this method improves the above non-zero mean shortcomings, the effect is similar to the maximum and minimum normalization, but in this study, in order to make the data can be amplified while maintaining positive values, so choose to treat the data with the maximum and minimum normalization, the deterioration curve after normalization is shown in Figure S4.

$$X_{norm} = \frac{X - X_{min}}{X_{max} - X_{min}} \in [0, 1] \cdots\cdots \qquad 6$$

$$X_{norm} = \frac{X - \mu}{X_{max} - X_{min}} \in [-1, 1] \cdots\cdots \qquad 7$$

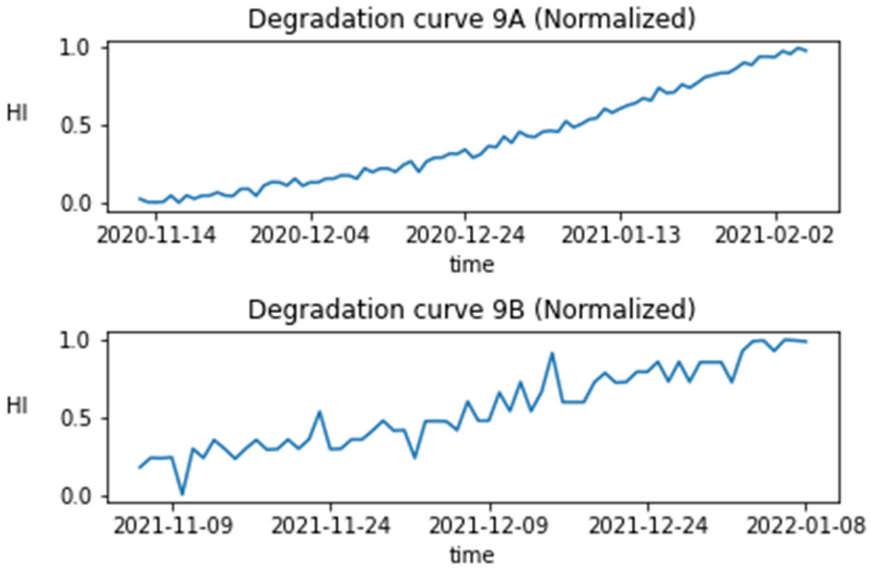

Figure S4. Deterioration curve after regularization (9A/9B).
